# Relationship between AZFc deletions and testicular histology in infertile South Chinese men with azoospermia and severe oligospermia

**DOI:** 10.1186/s40064-016-3512-7

**Published:** 2016-10-18

**Authors:** Quan Li, Ning-Hong Song, Wen-Zhou Cao, Qiang Shao, Jian-Jun Xie, Chao Liu, Ya-Min Wang, Hua Shen

**Affiliations:** 1 Department of Urology, Suzhou Municipal Hospital Affiliated to Nanjing Medical University, 16 Baita Road, Suzhou, 215001 China; 2 Department of Urology, First Affiliated Hospital of Nanjing Medical University, Nanjing, 210029 China

**Keywords:** Male infertility, FNAC, Testicular phenotype, Spermatogenesis, AZFc partial deletion

## Abstract

**Background:**

The AZFc deletion has been associated with wide range of phenotypes including complete absence of germ cells in the testes (SCOS), reduction in germ cells hypospermatogenesis, and maturation arrest. The main objective of this study was to evaluate the relationship between AZFc microdeletions and testicular histology in South Chinese men with azoospermia or severe oligospermia.

**Findings:**

338 men presenting with idiopathic non-obstructive azoospermia or severe oligospermia were evaluated between March 2012 and April 2015. Thirty-nine of the patients examined had an AZFc deletion (10.9 %). Testicular cytopathology was examined in 25 patients with an AZFc microdeletion and 14 with an AZFc deletion. There was no significant difference in the testicular histology of patients with partial or complete AZFc deletions (Mann–Whitney U = 152.500, p = 0.515). There was an association between testicular histology and gr/gr, b1/b3 or b2/b3 deletion (Fisher’s exact test, p = 0.013).

**Conclusions:**

Men with a gr/gr partial deletion were at higher risk of having hypospermatogenesis or maturation arrest. Men with a b1/b3 partial deletion were at higher risk of having maturation arrest. Men with a b2/b3 partial deletion were at higher risk of having maturation arrest or complete absence of germ cells in the testes.

## Background

The azoospermia factor (AZF) region on chromosome Yq involves three nonoverlapping loci -AZFa, AZFb, and AZFc. Microdeletions in this area are associated with spermatogenic failure and abnormalities in male germ cell development and maintenance (Vogt et al. 1996; Kuroda-Kawaguchi et al. 2001). Of infertile patients with Y chromosome deletions, 5 % have deletions in AZFa, 10–16 % have deletions in AZFb, and 60 % have deletions in AZFc (Vogt 1998; Foresta et al. 2001; Hopps et al. 2003). Deletions in two or three AZF regions have been reported in 14 % of infertile patients (Foresta et al. 2001). Deletion of the entire AZFa region is associated with the severe testicular phenotype, Sertoli cell only syndrome. Complete removal of the AZFb region is associated with spermatogenic arrest at meiosis (Vogt et al. 1996; Dada et al. 2004).

The most frequent deletion of the Y chromosome (AZFc, b2/b4) spans 3.5 Mb and eliminates 21 genes and a transcription unit in the AZFc region. Among the genes lost are four copies of the testis specific gene, DAZ (deleted in azoospermia). DAZ encodes for an RNA binding protein (Vogt et al. 1996; Kuroda-Kawaguchi et al. 2001). The AZFc deletion has been associated with wide range of phenotypes including complete absence of germ cells in the testes (SCOS), reduction in germ cells hypospermatogenesis (HP), and maturation arrest (MA). These findings suggest that the AZFc genes play a role in maturation of post-meiotic germ cells or spermatozoa (Vogt et al. 1996; Ferlin et al. 1999; Ferrás et al. 2004).

Expression profiling of the human testis has been widely used in the identification of genes involved in different key steps of testis development and function (Ostermeier et al. 2002; Massart et al. 2012). We evaluated 338 men with idiopathic infertility and identified 165 with secretory azoospermia (SAZ) and 173 with severe oligospermia (SOZ). These men were screened for partial AZFc deletions using AZFc-STSs. Examination of testicular histology was also performed. The aim of this study was to describe the relationship between AZFc partial deletions and the testicular histology of Southern Chinese men.

## Patients and methods

### Patients

338 men presented with idiopathic non-obstructive azoospermia (secretory azoospermia) or severe oligospermia with a sperm count less than 1.0 million/ml, between March, 2012 and April, 2015. General clinical data and blood samples were obtained from the center for Reproductive Medicine and Urology, First Affiliated Hospital of Nanjing Medical University, and Suzhou Municipal Hospital.

Exclusion criteria were a history of drug consumption, fever in the previous 6 months, seminal vesical infection, varicocele, systemic disease, previous cryptorchidism or orchitis, presence of anti-sperm antibodies, hypogonadotropic hypogonadism, abuse of androgenic steroids, treatment with chemotherapeutic agents or radiotherapy, testicular tumors or karyotypic abnormalities (Ferlin et al. 2006). Semen analysis was performed according to the World Health Organization guidelines on at least two occasions separated by three months (World Health Organization 2001). The diagnosis of azoospermia was established by pellet analysis after semen centrifugation.

This study was approved by the ethics committee of the Suzhou Municipal Hospital and First Affiliated Hospital of Nanjing Medical University. All participants provided informed consent.

### AZFc-STS analysis

**Fig. 1:**
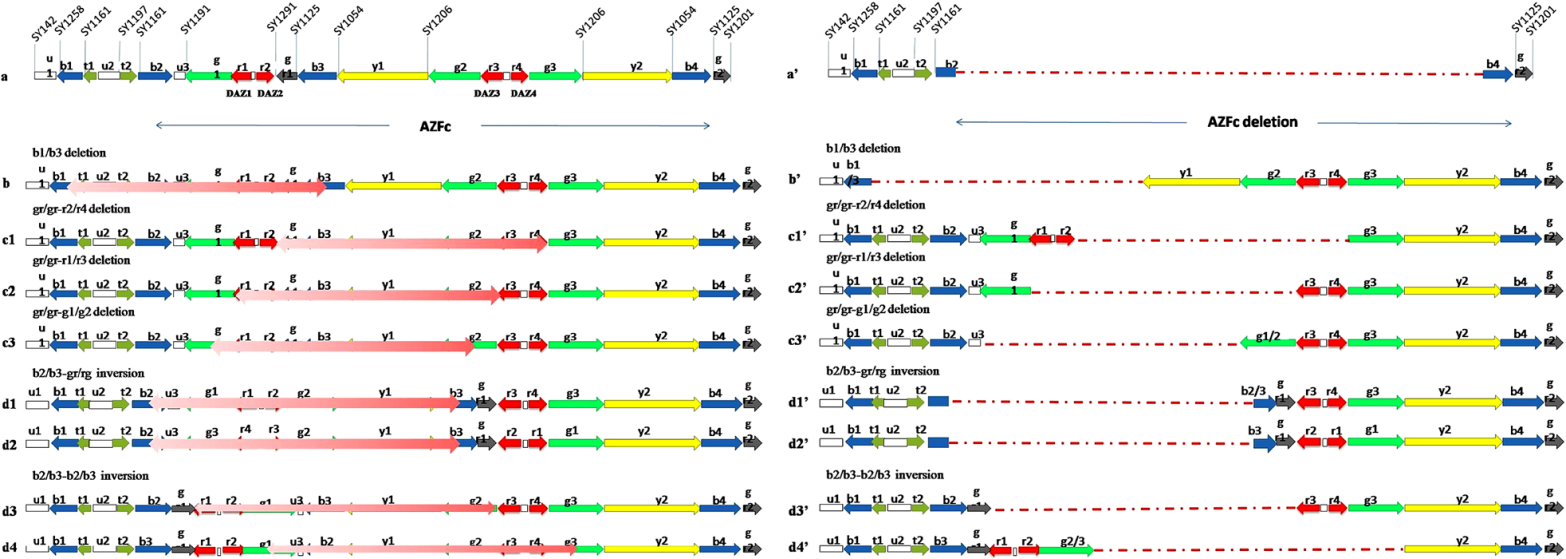
Schematic representation of the AZFc region of Chromosome Y, showing amplicon arrangements. STSs, and the rearrangements of AZFc deletions and microdeletions. **a** Reference sequence of the AZFc region with STSs used for detecting deletions in AZFc. STSs are shown above the AZFc sequence line. Arrows represent different amplicons and their orientation. **a** Derived from Kuroda-Kawaguchi [2]. *Red copies* numbered **r1** to **r4** show the position of the four DAZ copies. **a**′ The amplicon structure of the AZFc region with complete **b2/b4** deletion is shown. A **b2/b4** DAZ deletion was defined as the presence of sY1201, sY1161 and sY1125, and loss of sY1191, sY1291, sY1054 and sY1206. **b**, **b**′ A **b1/b3** deletion was defined as loss of sY1161, sY1191 and sY1291 and the presence of other STSs. Three different **gr/gr** deletions of the AZFc region were observed in **c1**, **c2**, **c3** and **c1**′, **c2**′, **c3**′. These deletions were defined as loss of sY1291 and the presence of the other STSs: (1) **c1** (**c1**′) shows a DAZ3/DAZ4 deletion, and (2) **c2** (**c2**′), (3) **c3** (**c3**′) show a DAZ1/DAZ2 deletion. Several **b2/b3** partial deletions are shown in **d1**, **d2**, **d3**, **d4** and **d1**′, **d2**′, **d3**′, **d4**′. A **b2/b3** deletion was defined as loss of sY1191 and the presence of the other STSs. Two mechanisms for these deletions have been reported: (1) **gr/gr** inversion (**g1**, **r1**, **r2** recombination with **r3**, **r4** and **g3**) followed by **b2/b3** deletion via homologous recombination (**d1** and **d2**) and (2) **b2/b3** inversion followed by **rg/rg** deletion via homologous recombination (**d3** and **d4**)

Genomic DNA was extracted from peripheral blood lymphocytes using DNA-isolation kits (TaKaRa Co, Ostu, Japan). Eight AZFc-specific STSs (sY116, sY1191, sY1197, sY1291, sY1125, sY1054, sY1206 and sY1201) were used to detect partial AZFc deletions (Simoni et al. 2004; Fukushima et al. 2006) (Fig. 1). ZFX/Y was amplified with other STSs as a positive internal control and DNA from a woman was used as a negative control. The primer sequences and PCR conditions were described previously (Simoni et al. 2004; Repping et al. 2003, 2004). PCR products were separated on a 2 % agarose gel and visualized using ethidium bromide staining. All samples were tested twice. STS amplification patterns that reflect AZFc deletions and AZFc microdeletions are shown in Fig. 1.

### Testicular histology

Fine-needle aspiration cytopathology (FNAC) was performed on patients with AZFc deletions or AZFc partial deletions and SAZ or SOZ. Bilateral testicular FNAC was performed as previously described (Bettella et al. 2005; Srivastava et al. 2004). Testicular phenotype was classified as follows (1) SCOS: presence of Sertoli cells only; (2) HP: all cell types up to spermatozoa were present, but there was a marked decrease in the number of reproducing spermatogonia; or (3) MA: incomplete spermatogenesis, limited to the spermatocyte stage (McLachlan et al. 2007).

### Statistical analysis

SPSS 11.0 (SPSS Inc, Chicago, IL, USA) was used for statistical analyses. The distributions of gr/gr, b2/b3 and b1/b3 partial deletions were compared between men with SAZ and SOZ using the Chi squared test. The relative frequency of single AZFc partial deletions in men with SAZ and SOZ was evaluated using Fisher’s exact test. All statistical tests were two-sided. p less than 0.05 was considered statistically significant.

## Results

### Patient general characteristics

338 patients with primary infertility were evaluated. 165 presented with SAZ and 173 with SOZ. All azoospermic patients had a non-obstructive aetiology on standard clinical evaluation. The mean age of the patients with SAZ was 26.3 years (range 21–40 years) and the mean age of patients with SOZ was 25.4 years (range 22–37 years). All patients had a normal 46, XY karyotype.

### Frequency of AZFc deletions and microdeletions in Southern Chinese men

Of the 338 patients examined, 39 (39/338, 11.54 %) had complete AZFc deletions or partial deletions (gr/gr, b1/b3, or b2/b3). Twenty-five of 39 (25/338, 7.40 %) had AZFc partial deletions and 14 (14/338, 4.14 %) had complete AZFc deletions. Eighteen of these 39 patients had SAZ (10 AZFc partial deletions, and 8 AZFc deletions) and 21 had SOZ (15 AZFc partial deletions and 6 AZFc deletions) (Table 1). AZFc partial deletions were defined as gr/gr (BPY2-BPY2, including DAZ1/2 or DAZ3/4), b1/b3 (RBMY1-, including DAZ1/2 or DAZ3/4). AZFc deletions were defined as b2/b4 (entire AZFc region) (Fig. 1).

**Table 1:** AZFc deletion analysis of peripheral leukocyte DNA from patients with idiopathic infertility

	Partial AZFc deletion			Complete AZFc deletion	Total N (%)
	gr/gr deletion (%)	b1/b3 deletion (%)	b2/b3 deletion (%)	b2/b4 deletion (%)	
Secretory azoospermia (*n*_A_ = 165)	5 (3.03)	2 (1.21)	3 (1.81)	8 (4.85)	18 (10.91)
Severe oligozoospermia (*n*_s_ = 173)	12 (6.94)	1 (0.58)	2 (1.16)	6 (3.47)	21 (12.14)
Total (*n*_T_ = 338)	17 (5.03)	3 (0.89)	5 (1.48)	14 (4.14)	39 (11.54)

Secretory azoospermia = no sperm in ejaculate; severe oligozoospermia = sperm count < 1 million/ml; n_A_ = the total number of secretory azoospermia; n_s_ = the total number of severe oligozoospermia

The frequency of AZFc partial deletions in SAZ and SOZ patients was not different (X^2^ = 1.061, p = 0.303) (Table 1). The frequency of AZFc deletions in SAZ and SOZ patients was also not different (X^2^ = 0.405, p = 0.524). The frequency of AZFc deletions plus AZFc partial deletions in SAZ and SOZ patients was not different (X^2^ = 1.061, p = 0.303).

### Testicular histology

Adequate material for histologic examination was obtained from all 39 patients. The smears were categorized as SCOS in 12 patients, MA in 19 patients, and HP in 8 patients. Eighteen of 39 (46.2 %) men had azoospermia and 21 (53.8 %) had oligozoospermia. Of the 25 patients with AZFc partial deletions, 7 had SCOS, 12 MA and 6 HP. Of the 14 patients with AZFc deletions, 5 had SCOS, 7 MA and 2 HP.

The patients with partial or complete AZFc deletions had no difference in testicular histology (Mann–Whitney U = 152.500, p = 0.474) (Table 2). The patients SAZ and partial or complete AZFc deletions had no difference in testicular histology (Mann–Whitney U = 39.000, p = 0.923). The patients SOZ and partial or complete AZFc deletions had no difference in testicular histology (Mann–Whitney U = 37.000, p = 0.495).

**Table 2:** Testicular histology of patients with partial or complete AZFc deletions by clinical presentation

	Partial AZFc deletion			Complete AZFc deletion			Total
	SCOS	MA	HP	SCOS	MA	HP	
Secretory azoospermia (*n*_A_ = 165)	4	4	2	3	4	1	18
Severe oligozoospermia (*n*_s_ = 173)	3	8	4	2	3	1	21
Total (*n*_T_ = 338)	7	12	6	5	7	2	39

Secretory azoospermia = no sperm in ejaculate; severe oligozoospermia = sperm count < 1 million/ml

*SCOS* sertoli cell-only syndrome, *MA* maturation arrest, *HP* hypospermatogenesis

### Correlation between AZFc deletions and testicular histology

The distribution of AZFc partial deletions and testicular histology by clinical presentation was examined (Table 3). Of the 12 patients with SCOS, four had gr/gr deletions, 3 b2/b3 deletions, and 5 b2/b4 deletions. Of the 19 patients with MA, 8 had gr/gr deletions, 3 b1/b3 deletions, 1 a b2/b3 deletion, and 7 b2/b4 deletions. Of the 8 patients with HP, 5 had gr/gr deletions, 1 a b2/b3 deletion, and 2 b2/b4 deletions.

**Table 3:** Testicular histology of patients with idiopathic infertility by location of AZFc deletion

	Testicular histology	Partial AZFc deletion			Complete AZFc deletion	Total
		gr/gr deletion	b1/b3 deletion	b2/b3 deletion	b2/b4 deletion	
Secretory azoospermia (*n*_A_ = 165)	SCOS	3	0	1	3	7
	MA	1	2	1	4	8
	HP	1	0	1	1	3
Severe oligozoospermia (*n*_s_ = 173)	SCOS	1	0	2	2	5
	MA	7	1	0	3	11
	HP	4	0	0	1	5
Total		17	3	5	14	39

Secretory azoospermia = no sperm in ejaculate; severe oligozoospermia = sperm count < 1 million/ml; n_A_ = the total number of secretory azoospermia; n_s_ = the total number of severe oligozoospermia

*SCOS* sertoli cell-only syndrome, *HP* hypospermatogenesis, *MA* maturation arrest

There was a significant difference in testicular histology by AZFc partial deletion (gr/gr, b1/b2 and b2/b3, p = 0.013, Fisher’s exact test). Of the 17 men with gr/gr partial deletions, 4 had SCOS, 8 MA and 5 HP. Of the 3 men with b1/b3 partial deletions, all had MA. Men with a gr/gr partial deletion were at higher risk of having HP or MA, men with a b1/b3 partial deletion were at higher risk of having MA, and men with a b2/b3 partial deletion were at higher risk of having MA or SCOS. Men with b2/b4 deletions (n = 14) were found to have either SOZ or SAZ (X^2^ = 0.405, p = 0.524).

## Discussion

Causes of testicular failure include genetic disorders (such as sexual chromosomal abnormalities, translocations and deletions of the Y chromosome), cryptorchidism, varicoceles, testicular torsion, radiation and toxins (Abbasi et al. 2011; Palermo et al. 1999). Testicular cytology is a crucial assessment tool in the evaluation of infertility and has prognostic importance for assisted reproductive technologies (ART). Men with complete AZFc deletions have clinical presentations ranging from azoospermia to severe oligospermia. Testicular phenotype in these patients includes maturation arrest and SCOS (Ferlin et al. 2007).

Infertile patients we examined with non-obstructive azoospermia and severe oligospermia had an increased prevalence of AZFc partial deletions (25/338, 7.40 %) and AZFc deletions (14/338, 4.14 %), suggesting these findings were related to their infertility. The greater frequency of partial deletions may better localize the source of this defect.

Partial deletions at b1/b3 and b2/b3 have been reported to have an impact on spermatogenesis (Wu et al. 2007). This variation may be attributed to the different ethnic and genetic backgrounds of the study subjects evaluated. This observation is corroborated by other studies (Krausz et al. 2009; Ferlin et al. 2005).

There were several limitations to this study. Only patients with infertility were examined. The prevalence of these genetic findings in the general population is not known. No germline analyses were performed to determine if these were inherited, somatic, or mosaic mutations. No family history of infertility was determined. Small, but potentially clinically significant deletions would not be detected using the methods employed here. The presence of so-called “micro” deletions could be much higher in the AZF regions.

Many studies found a positive correlation between AZFc microdeletions (gr/gr deletion and b2/b3 deletion) and spermatogenic failure, whereas other studies could not confirm the relationship between AZF microdeletions and male infertility (Ferlin et al. 2005; Visser et al. 2009). This disagreement may be due to different factors, such as ethnicity, country of study or geographic region (Stouffs et al. 2011).

This study provides further evidence that partial deletions of the AZFc region are a risk factor for decreased sperm quality. We found several partial deletions of this region to be associated with impaired spermatogenesis, suggesting multiple genes related to this process are located in this region. Partial deletions involving the DAZ and CDY clusters were associated with the clinical findings of SCOS, MA, and SOZ in the infertile patients we examined. Loss of different DAZ copies (DAZ1/2 or DAZ3/4) or CDY subtypes (CDY1a or CDY1b) were associated with these changes. Further work is needed to define the association between specific partial deletions of the AZFc region and clinical presentation.

## Conclusions

Previous studies indicate that men with AZFc partial deletions may naturally father children (Page et al. 1999). Those that required ART may have a risk of transmitting this mutation to their male children. Further studies of gr/gr, b1/b3 and b2/b3 deletions and specific gene loss are needed to better understand this phenomenon.
